# B-Mode and Doppler Ultrasonography in a Murine Model of Ehrlich Solid Carcinoma With Different Growth Patterns 

**DOI:** 10.3389/fonc.2020.560413

**Published:** 2020-11-04

**Authors:** Carla Martí Castelló, Marina Pacheco Miguel, Elisângela de Paula Silveira-Lacerda, Andris Figueiroa Bakuzis, Naida Cristina Borges

**Affiliations:** ^1^ Programa de Pós-Graduação em Ciência Animal, Escola de Veterinária e Zootecnia, Universidade Federal de Goiás, Goiânia, Brazil; ^2^ Setor de Patologia, Instituto de Patologia Tropical e Saúde Pública, Universidade Federal de Goiás, Goiânia, Brazil; ^3^ Instituto de Ciências Biológicas, Universidade Federal de Goiás, Goiânia, Brazil; ^4^ Instituto de Física, Universidade Federal de Goiás, Goiânia, Brazil

**Keywords:** Ehrlich solid carcinoma, tumor vascularization, ultrasound, breast cancer, comparative medicine, growth pattern

## Abstract

Ehrlich solid carcinoma (ESC) is one of the tumor models used in cancer research. Although it is widely used, it has no ultrasonographic descriptions. In this study, serial B-mode and Doppler ultrasonographic examinations were performed for 23 days for ESCs inoculated into 18 Swiss albino mice. The growth patterns were analyzed, and on the basis of their growth curve, the tumors were classified into two groups: fast growth (FG) and slow growth (SG). Ultrasonographic characteristics of the tumor’s capsule, margins, echogenicity, echotexture, vascular index (VI), distribution of vascular flow, and Doppler indices such as the resistive index, pulsatility index, and peak systolic velocity (SV) were analyzed and compared between the two groups. A high VI and earlier blood flow were noted in the FG group (p<0.05). Additionally, SV was higher in the FG group than in the SG group (13.28 ± 0.38 cm/s vs. 8.43 ± 0.26 cm/s). In contrast, a change in echogenicity and flow distribution patterns were observed, especially in FG tumors. Therefore, ESC presented with few ultrasonographic differences between FG and SG tumors, especially vascularization during the initial stages of tumor growth.

## Introduction

Cancer is one of the most common and serious diseases of the current clinical medicine state and one of the main causes of high mortality rates due to late diagnosis. Tumor models are tools of great importance in cancer research. *In vivo* murine models capture the complexity of neoplastic growth and metastatic process in a living system, but individually viewing the steps and quantitatively extracting data are generally difficult (1).

Mice models have been developed and their ability to model many diverse aspects of human diseases have been improved; a similar need to develop and improve imaging approaches to measure key biological parameters noninvasively exists. In this context, ultrasound imaging is especially useful in measuring the size, internal aspect, and characteristics of vascularity and blood perfusion in soft tissue tumor (2) For this reason, it has been used in this study and for the diagnosis of some types of human (3, 4) and canine cancers (5).

Imaging including magnetic resonance imaging and computed tomography has been used in rodents (6), dogs (7), and humans (8) for cancer research. Recently, tumors in rodents has also been studied by imaging methods that evaluate tumor microvasculature using contrast agents, like dynamic contrast-enhanced ultrasound (DCE-US) or acoustic angiography (6, 9, 10). Ultrasound imaging offers a noninvasive cost-effective analysis of anatomy and vascular perfusion and is compatible with most treatments. For example, Doppler ultrasonography is an extremely useful tool for the detection of blood flow changes following antivascular treatments (11).

Ehrlich carcinoma is a tumor model largely used in cancer research. It originates from spontaneous murine mammary adenocarcinoma and adapts to an ascites form by intraperitoneal serial passages (12). The solid form i.e., Ehrlich solid carcinoma (ESC) is generated from subcutaneous inoculation and considered an important tool in the investigation of antineoplastic treatments (13, 14). Although ESC is being used in thousands of studies annually, especially for testing new antineoplastic treatments and their toxicity (15, 16), there are few studies on their biology, and we did not find published studies on its ultrasonographic aspect.

Therefore, this study aimed to describe the B-mode and Doppler ultrasonographic examinations of ESC inoculated in mice and study the growth kinetics using size measurements obtained with ultrasound, relationship between growth patterns, morphological aspects, blood flow characteristics, and Doppler indices. Besides this, ultrasound is demonstrated as a tool that can help in the study of new treatments using ESC.

## Materials and Methods

### Animals

The study was part of an integrated research project approved by the Animal Use Ethics Committee with protocol number 098/14.

In this study, a total of 18 male Swiss albino mice with body weight between 25 and 40 g and age between 6 and 8 weeks at the time of inoculation were analyzed. The animals were maintained under standard laboratory conditions i.e., 22°C–25°C with a dark/light cycle of 12/12 h and allowed free access to a standard dry pellet diet and water ad libitum. The mice were anesthetized using a 0.2 ml/100 g dose of a solution containing 100 mg/ml of 10% ketamine and 12.5 mg/ml of 2% xylazine. The solution was injected intraperitoneally, and the mice were euthanized by cervical dislocation. The procedures involving the management and care of the animals were based on the Guide for the Care and Use of Laboratory Animals (17) to minimize the pain of animals by incorporating the correct procedures.

### Murine Tumor Model and Experimental Design

To generate solid tumors, Ehrlich ascites carcinoma was injected in Swiss mice intraperitoneally. Seven days after cell inoculation, the peritoneal fluid of a mouse with Ehrlich ascites carcinoma was aspirated, the cells were washed using sterile PBS, and an aliquot of the cell suspension was diluted in trypan blue 1% (m/v) (Sigma, St. Louis, MO) and quantified using the Luna automated cell counter (Logos Biosystems, Annandale, VA). Only cell dilutions containing 90% of viable cells were injected into the subcutaneous tissue of the left scapular region of the 18 mice to induce ESC. The mice were grouped according to their growth pattern. Animals that showed palpable and ultrasonographically measurable tumor growth from day 5 after inoculation were included in the fast growth (FG) group and from day 10 after inoculation in the slow growth (SG) group.

### Ultrasonographic Study

The first ultrasonographic examination was performed on each animal on the day of tumor induction. Other ultrasound examinations were performed on days 5, 10, 12, 14, 16, 18, 20, and 23 after tumor induction.

**Figure 1:**
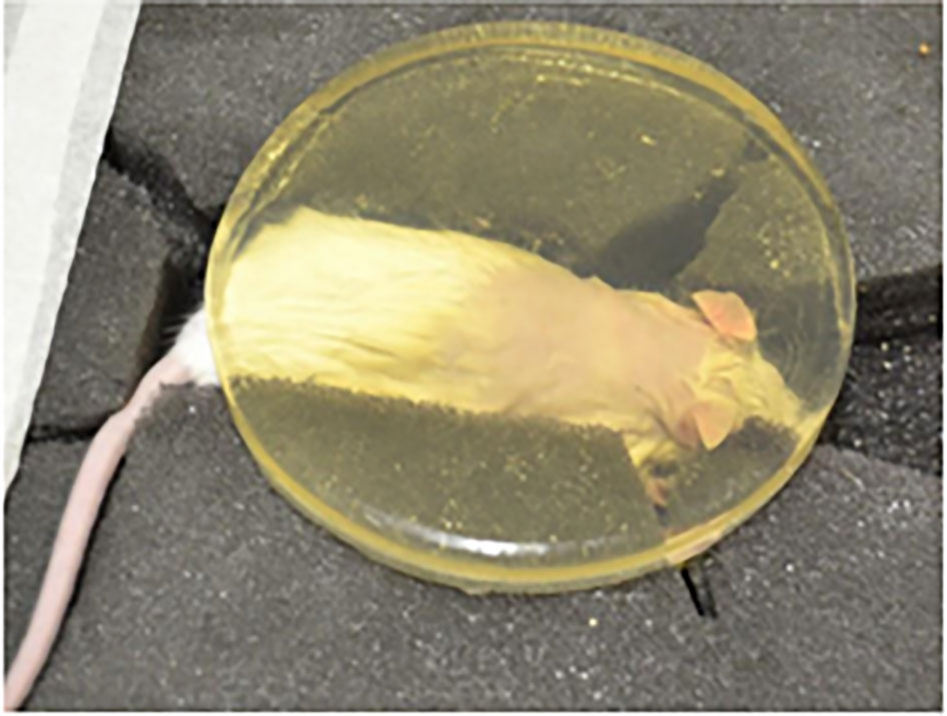
A Swiss albino mouse in ventral recumbence in the foam bed, with the acoustic solid gelatin on him.

The mice were anesthetized and accommodated in ventral recumbency on a polyurethane foam bed, especially designed for that purpose (**Figure 1**). After evaluation, the Ehrlich induced subcutaneous tumors were examined by ultrasound with 18-MHz linear transducer (MyLab 30, Esaote, Genoa, Italy). Using imaging settings for small body parts as a reference, we created a specific preset for subcutaneous tumor models in mice: depth 3 cm, gain B-mode 52%, and dynamic range 10. Two focal areas were standardized.

**Figure 2:**
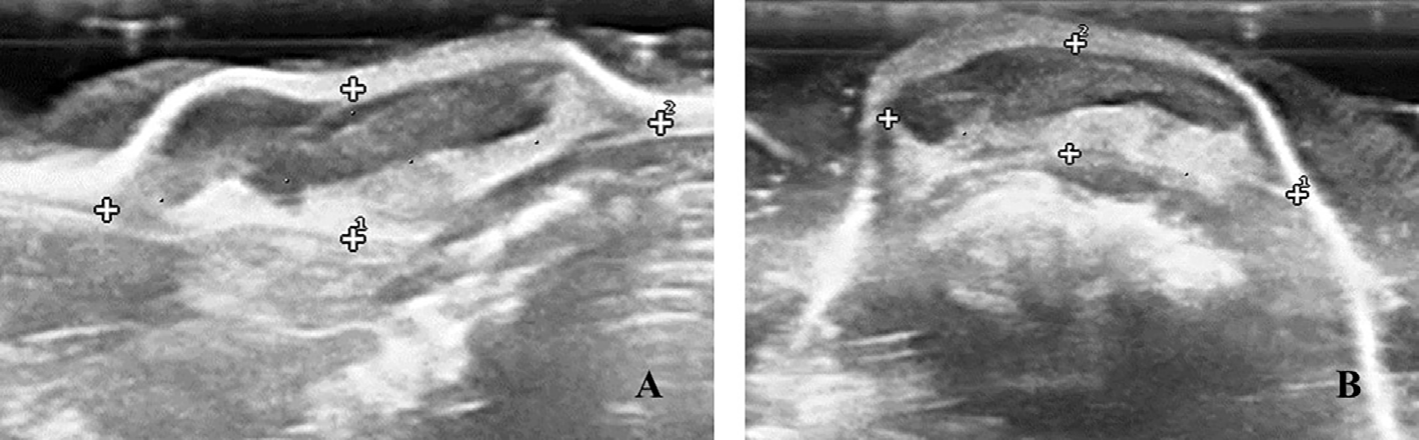
B-mode ultrasound images of Ehrlich Solid Carcinoma induced in the subcutaneous tissue of Swiss albino mouse, showing in **(A)** Longitudinal view showing the measurements of length and height. **(B)** Cross-sectional view showing width and height measurements. 18 MHz transducer.

The ultrasonographic features evaluated using B-mode were volume, capsule, echogenicity, and margins (18–20). Longitudinal and transversal views were obtained from each tumor to calculate the volume using the empiric formula of Lambert: length (L) × width (W) × height (H) × 0.71 (**Figure 2**) (19). The specific growth rate (SGR) was calculated with the formula SGR = ln (V_2_/V_1_)/(t_2_ – t_1_), and the doubling time (DT) was calculated with the formula DT = ln2/SGR = (t_2_ – t_1_) ln2/ln (V_2_/V_1_), where V_2_ is the final volume, V_1_ is the initial volume, t_2_ is the final time, and t_1_ is the initial time (21).

Margins of the tumors were classified as defined or undefined depending on whether the margin between the tumor and surrounding normal tissue was distinct or not (19). A capsule was considered present when the tumor had already more than one image with the most part around the tumor with a hyperechoic line clearly around it. Tumor echogenicity was compared to the surrounding tissue echogenicity and it was classified as hypoechoic, hyperechoic, or mixed (20). Echotexture was classified as homogeneous or heterogeneous (19, 20), although interpretation of echotexture is subjective, it has been proposed than echotexture is homogeneous when there was an uniform layer of tissue or tiny hipoechoic areas were mixed uniformly in the tumor and heterogeneous when the tumor had multiple areas with increased or decreased echogenicity (22).

Doppler examination was performed using acoustic solid gelatin, which had similar functions and characteristics as a commercial standoff pad. The aim of using this acoustic solid gelatin was to prevent echo reverberations in the area of interest by placing it in the focal zone of the transducer (**Figure 1**) (23).

Color flow mapping was used in different planes to evaluate the vascular supply in the tumor and peripheral tissues. Color gain was adjusted to reduce excessive color noise when the blood flow was too slow and the pulse repetition frequency was 2.1 kHz. The number of vessels in each plane was quantified, and the vascularity index (VI) (number of vessels/area in cm^2^) was calculated with an average of three or more vascularized planes (11). When vascular supply was detected, the distribution of vascular flow was classified into three flow patterns: central, peripheral, and diffuse (central and peripheral) (19).

Doppler analysis was performed while keeping the angle between the Doppler beam and long axis of the vessel (θ) < 60°. The Doppler gate was established at 2 mm. The transducer was manipulated to optimize the spectral waveform. The resistive index (RI), pulsatility index (PI), peak systolic velocity (SV), and middle velocity (mV) were measured using onboard software as follows: RI = (SV - end diastolic velocity)/SV; PI = (SV - end diastolic velocity)/time-averaged maximum velocity. Three measurements were obtained for each parameter in various locations within the tumor, and the average of these measurements was used in the analysis. We recorded and stored the images digitally for subsequent analysis and calculations.

### Statistical Analysis

The unpaired t-test was conducted to compare the parametric variables volume, VI, Doppler indices (RI, PI, SV), and DT between the two groups. To evaluate the independence of the groups, the Fisher’s Exact test was conducted for morphology variable margins, echotexture, echogenicity and capsule, and the chi-square test was conducted for flow distribution. All analyses were performed using GraphPad Prism (Prism 6 for Windows, version 6.01, 2012).

## Results

We calculated the tumor volume at each evaluation moment from the mean value of at least three measures of longitudinal diameter, transverse diameter, and depth. On the first ultrasound evaluation, no animal showed signs of tumor or any difference to animals without tumor induction. Five days after induction, only eight animals (44%) presented growth, with volumes between 199 and 441 mm^3^. These animals comprised the FG group. It was not until day 10 that other tumors grew (volumes between 164 and 393 mm^3^), and these 10 animals comprised the SG group. Comparing the tumor volumes in all examinations between the groups, a statistically significant difference was noted (**Table 1**).

**Table 1:** Comparison of B-mode data between the groups.

Variables	B-mode Ultrasonography			
	Parameter	Fast Growth	Slow Growth	p-value
**Volume (mm^3^)**	Mean value	1539 ± 75.4	639.4 ± 34.2	<0.0001*
**Doubling Time (days)**	Mean value	3.57 ± 0.20	7.31 ± 0.53	<0.0001*
**Capsule (%)**	Present	75	90	0.2745
	Absent	25	10	
**Echotexture (%)**	Homogeneous	34.4	74.3	<0.0001*
	Heterogeneous	65.2	25.7	
**Echotexture ISV (%)**	Homogeneous	100	100	1.0000
	Heterogeneous	0	0	
**Echotexture LSV (%)**	Homogeneous	50	50	1.0000
	Heterogeneous	50	50	
**Echogenicity (%)**	Hypoechoic	7.8	32.9	0.0005*
	Mixed	92.2	67.1	
**Echogenicity ISV (%)**	Hypoechoic	37.5	80	0.1448
	Mixed	62.5	20	
**Echogenicity LSV (%)**	Hypoechoic	12.5	10	1.0000
	Mixed	87.5	90	
**Margins (%)**	Defined	40.6	65.5	0.0001*
	Undefined	59.4	34.5	
**Margins ISV (%)**	Defined	62.5	100	0.0686
	Undefined	37.5	0	
**Margins LSV (%)**	Defined	50	55	1.0000
	Undefined	50	45	

For variables Doubling Time and Capsule, we have only one data for each tumor. For variables Volume, Echotexture, Echogenicity and Margins, we used de data of all moments except when we stipulate to compare Initial Similar Volumes (ISV). To compare de variables Echotexture, Echogenicity, Capsule and Margins between groups was conducted an exact Fisher test and for variables Volume and Doubling Time an Unpaired t-test, for all of them we considered statistical significance with a p-value < 0.05. *statistical significance.

**Figure 3:**
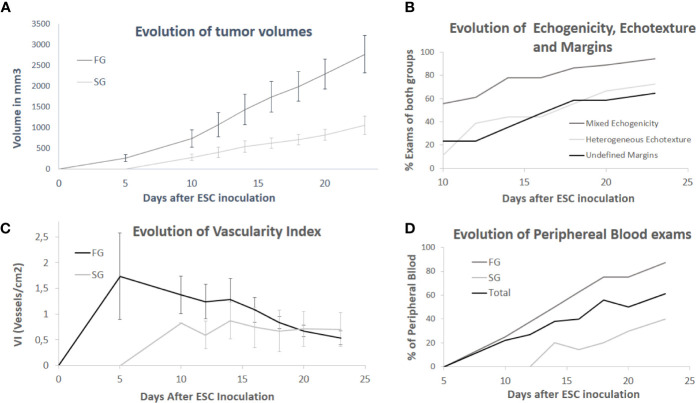
Graphics showing the evolution during 23 days of some variables of two groups of Swiss Albino mice inoculated with Ehrlich Solid Carcinoma with different tumor growth pattern. **(A)** The mean of volumes with standard deviation. **(B)** Percent of exams showing mixed echogenicity, homogeneous echotexture and undefined margins. **(C)** Vascularity Index. **(D)** Percent of exams with peripheral blood flow.

The kinetics of growth was analyzed using a graphic image (**Figure 3A**). Two linear functions, in which the FG function was increasing faster than the SG function, was observed. In contrast, we analyzed the volume daily; DT and significant differences were observed between the FG and SG groups (**Table 1**). When we considered the tumor volumes daily, we detected differences, but when we compared the first examination of each group, there were no significant differences (p=0.6107). The same phenomenon occurred when we compared the days of largest similar volumes (LSV) (day 14 of FG with day 23 of SG) (p=0.9292). Thus, day 5 of FG and 10 of SG were considered initial similar volumes (ISVs) and day 14 of FG and 23 of SG were considered LSVs.

**Figure 4:**
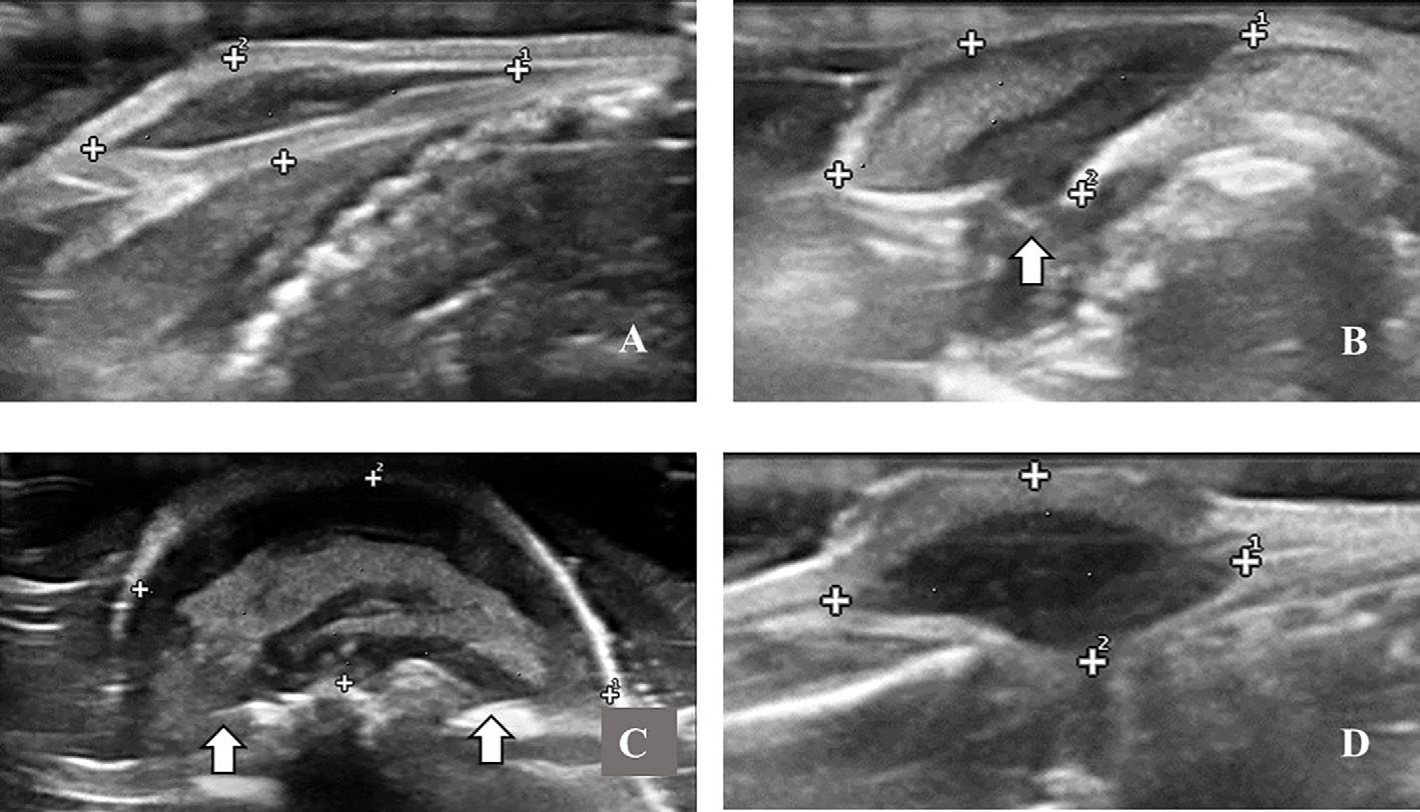
B-mode ultrasound images of Ehrlich Solid Carcinoma induced in the subcutaneous tissue of Swiss albino mice; showing in **(A)** complete capsule and circumscribed margins; **(B)** discontinuity in the capsule (marked by arrow), giving rise to an image with partially undefined margins, **(C)** significant discontinuity in the capsule (marked by arrows), giving rise to an image with indistinct margins, evidencing too, the heterogeneous interior; **(D)** homogeneous and hipoechoic interior.

By analyzing the morphology variables, we considered 77.8% of the tumor with the capsule (**Figure 4A**). Sometimes, the capsule presented discontinuity in some images (**Figure 4B**). In 58.2% of ultrasound evaluations, the tumors obtained defined margins, and in 55.2% homogeneous echotexture. Considering echogenicity, it was found that 20.9% of examinations presented as hypoechoic (**Figure 4D**) and 79.1% mixed echogenicity (**Figure 4C**).

The B-mode ultrasonographic variable margins, echotexture and echogenicity showed differences between the groups (**Table 1**). In contrast, when we compared daily and similar volumes we found differences in some days but never between similar volumes (**Table 1**). This suggests that the difference observed can be because of the difference in volume between the groups. We also noted that there was a tendency to increase the percentage of tumors with heterogeneous echotexture, mixed echogenicity and undefined margins over time (**Figure 3B**).

**Figure 5:**
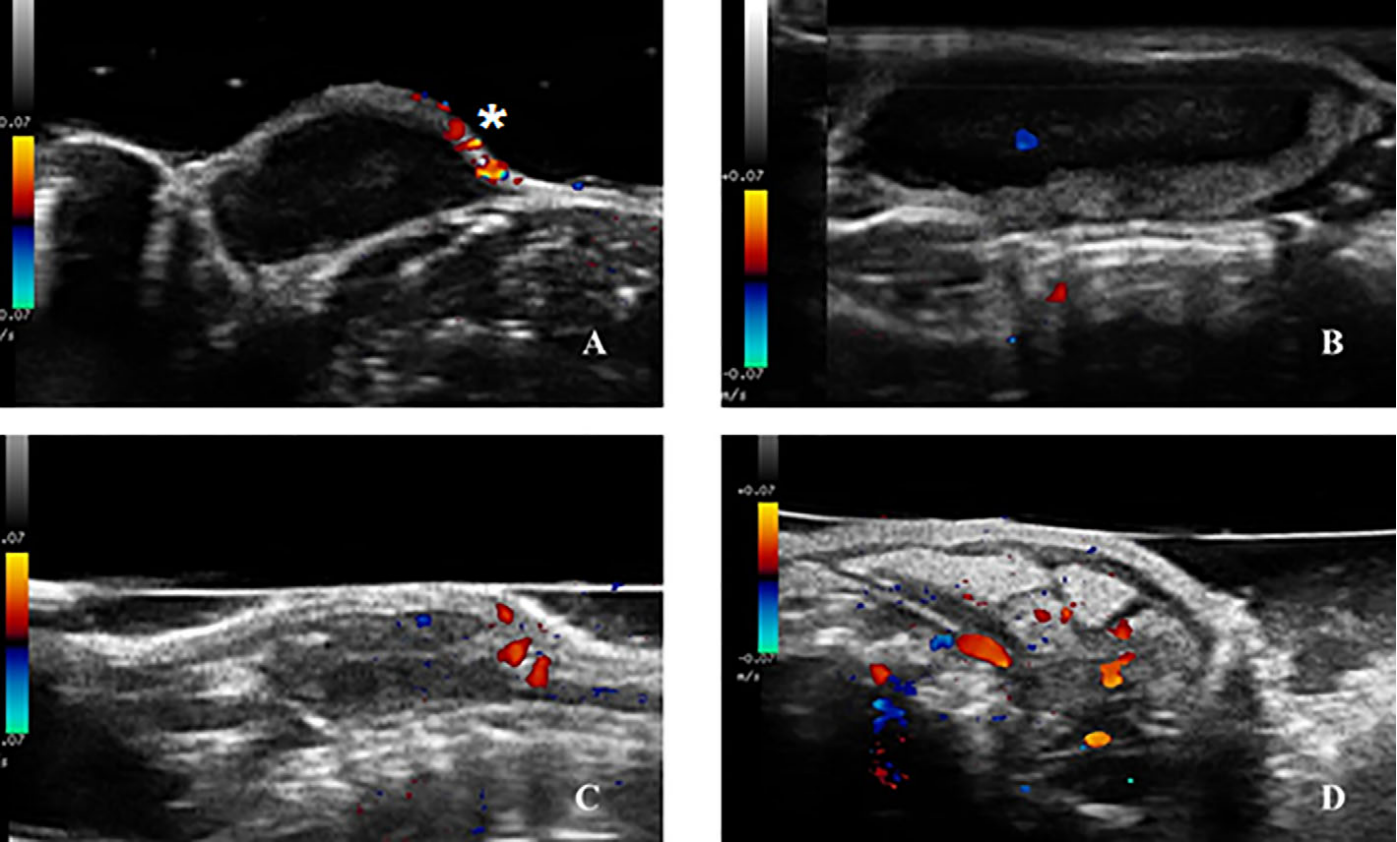
Color Doppler mode ultrasound images of Ehrlich Solid Carcinoma induced in the subcutaneous tissue of Swiss albino mice; showing in **(A)** tumor without vascular flow, the signals of flow in the skin marked with (*) are not considered tumor flow; **(B)** image with a single intratumoral vessel with central distribution flow pattern; **(C)** image with four intratumoral vessels with peripheral distribution flow pattern; **(D)** image with more than five intratumoral vessels with mixed distribution flow pattern.

Color flow mapping was used to study the presence and absence of blood flow, VI, and localization of vessels (**Figure 5**). All tumors presented detectable blood flow in at least three examinations. Considering the examinations individually, 19.4% did not present blood flow, while 80.6% did. Only 19.4% of the examinations did not show blood flow, but 12 animals (66.7%) did not present flow in at least one examination, nine of which belonged to the SG group and presented this absence of flow in the first ultrasound examinations. In contrast, there were six animals (33.3%) that presented flow in all examinations, five of which were part of the FG group. We observed differences between the groups until day 14 or ISV, but no differences were detected after day 14 or LSV (after day 18, all examinations had flow). This indicates that the presence of flow in the initial phases of ESC growth could be an important characteristic for predicting growth.

The average volume during the first blood flow was 485 mm^3^, and no difference was observed between the groups. The mean duration between the first examination of tumor appearance and the first examination of blood flow detection was 3.39 days, and a difference was observed between the groups (p=0,0447; FG: 1.87 ± 0.91 and SG: 4.6 ± 0.85). Hence, detectable blood flow in tumors first presented in the FG group than in the SG group.

If we disregarded examinations with no blood flow, the highest detected VI was 2.58 vessels/cm^2^, the lowest was 0.16, and the mean value was 0.91 ± 0.04. There were differences between groups when we compared all days (**Table 2**), which is significant because VI is an independent variable of volume, showing that FG tumors were more vascularized. On the contrary, if we compare the evolution of VI, we can regard that, in SG tumors, it was quite stable, while, in FG tumors, we found a large initial vascularization that decreases over time, being more pronounced after day 14 (**Figure 3C**). From day 12, there were no differences in VI between groups, and even in days 20 and 23, the FG group had a lower rate than the SG group (**Figure 3C**).

**Table 2:** Comparison of Doppler mode data between the groups.

Variables	Doppler Ultrasonography			
	Parameter	Fast Growth	Slow Growth	p-value
**Vascularization (%)**	Present	94.4	67.1	0.0002*
	Absent	5.6	32.9	
**Vascularization ISV (%)**	Present	62.5	10	0.0430*
	Absent	37.5	90	
**Vascularity Index (vessels/cm^2^)**	Mean value	1.064 ± 0.06	0.719 ± 0.05	<0.0001*
**Flow Localization (%)**	Central	8.2	17	0.6127
	Peripheral	54.1	29.8	
	Diffuse	37.7	53.2	
**Systolic Velocity (cm/s)**	Mean value	12.60 ± 0.27	8.52 ± 0.31	<0.0001*
**Resistive Index**	Mean value	0.64 ± 0.006	0.61 ± 0.007	0.3238
**Pulsatility Index**	Mean value	1.14 ± 0.023	1.11 ± 0.025	0.3779

In all variables, we used de data of all moments except when we stipulate to compare Initial Similar Volumes (ISV) or Largest Similar Volumes (LSV). To compare de variable presence of Vascularization between groups was conducted an exact Fisher test, for variables Vascularity Index, Systolic Velocity, Resistive Index and Pulsatility Index an Unpaired t-test and for variable Flow Localization a chi-square test, for all of them we considered statistical significance with a p-value <0.05. *statistical significance.

Color flow mapping was also used to study the blood flow distribution. Considering only the examinations with positive flow, the flow was central in 12.04%, (**Figure 5B**), peripheral in 43.52% (**Figure 5C**), and mixed in 44.44% (**Figure 5D**). We observed that, in the initial stages (until day 16), most neoplasms had diffuse flow distribution, while, in more advanced stages (from day 18), they were mostly peripheral, showing an increase in tumors with peripheral flow distribution over time (**Figure 3D**). The chi-square test was performed to correlate the frequency of different types of blood flow distribution between the FG and SG groups; there were no differences (**Table 2**).

**Figure 6:**
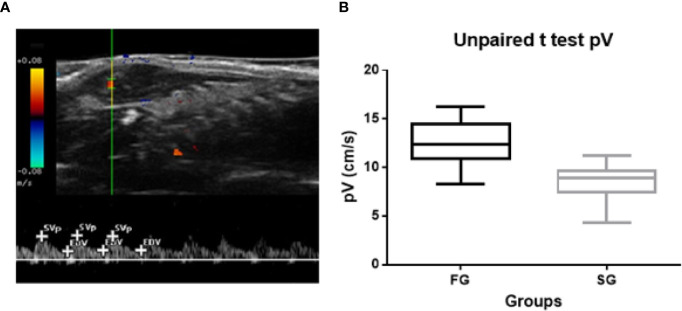
**(A)** Triplex Doppler mode ultrasound images of Ehrlich Solid Carcinoma induced in the subcutaneous tissue of Swiss albino mice; showing an intratumoral vessel and their spectral trace showing the minimum of three consecutive waves marking the peak of systolic velocity and the end of diastolic velocity (+ cursors). **(B)** Graphic showing the statistical differences of peak Systolic Velocity between groups (p < 0.0001).

The RI ranged from 0.52 to 0.79, with a mean value of 0.63 ± 0.049; the PI ranged from 0.64 to 2.75, with a mean value of 1.27 ± 0.3856; and the SV ranged from 4.3 to 20.83 cm/s, with a mean value of 11.25 ± 3.37. Student’s t-test was performed, and a correlation between SV and groups separated by growth curve was shown (p<0.0001). The mean SVs were 13.28 ± 0.38 cm/s in the FG group and 8.43 ± 0.26 cm/s in the SG group. Therefore, a tumor with SV > 12.89 cm/s is more likely to belong to the FG group and that with SV < 8.69 cm/s to the SG group (**Figure 6**).

## Discussion

A tendency toward heterogeneous echotexture, mixed echogenicity, undefined margins, and peripheral vascular pattern were observed in ESC inoculated in Swiss albino mice with increased volume. FG tumors are related to earlier presence of vascularization, higher VI, and higher SV, so we can consider these parameters to predict growth pattern. Thus, we can deduce that vascularization during the early stages is of great importance for rapid tumor growth, as observed in mice ultrasound findings.

In the present study, ESC presented different growth patterns. Studies on the growth and behavior of Ehrlich ascites carcinomas show different rates in various strains of mice, and inflammatory mechanisms are relevant in tumor-host interactions in this model (24). Although the role of inflammatory and immune responses is complex, it is well known that there is an important relationship between immune system responses and tumor development (25).

Although all mice in the study had the same genetic lineage, they may have intrinsic characteristics that have made a difference in their immune system. Extrinsic factors could also explain differences in the immune system that could have led to these differences in growth. Even though they were kept under the same conditions during the study, the animals of the two groups were raised at different times. The eight animals in the FG group may have had some stressful development that affected their immune system.

Previous studies showed tumor growth seven days after inoculation and a growth pattern similar to those in our study if we consider the average of the groups (12, 26). In contrast, few studies showed earlier and faster growth (13) similar to that in the FG group of our study, whereas others showed later and slower growth (14) or higher DT (27). These differences highlight the importance of maintaining all animals under similar conditions, especially in studies that compare the role of the immune system between control and treated groups.

In this study, ESC was observed by B-mode ultrasonography, such as tumors with a hyperechoic capsule and different characteristics depending on the tumor volume or stages of growth. During the early stages of growth, they are observed normally with circumscribed margins and hypoechogenic homogeneous echotexture, but at more advanced stages, larger volumes tend to appear with undefined margins and heterogeneous echotexture with parts the same echogenicity of the capsule and other hypoechogenic or anechogenic parts. This description can be compared with histopathological descriptions. Previous studies described ESC as surrounded by a well-defined fibrous capsule (12, 27), which, in large tumors, lose their integrity and present invasion of adjacent structures (13, 14, 28). The inside of the ESC has been described with a large necrotic center or with central necrotic areas around which there is a shell of viable tissue (12, 27).

Ehrlich carcinoma is a spontaneous mammary gland adenocarcinoma originating from the breasts of female mice with similar morphological and kinetic alterations to those occurring in human and canine breast cancers (29, 30). Hence, their ultrasound characteristics can be compared with those in studies of ESC biology and human and canine breast cancer ultrasonography reports.

A higher prevalence of mixed echogenicity in malignant tumors was shown in earlier reports (20, 31), but a study comparing different grades of a malignant neoplasm did not find differences between low and high grade (32). Echotexture differences were also not found among tumors of different grades (33). Another study showed a homogeneous hypoechoic aspect in mammary gland neoplastic lesions of small size (34).

In the present study, all tumors were ESC, a malignant neoplasm, and a high prevalence of mixed echogenicity was observed between the FG and SG groups, but just in the FG group was observed high prevalence of heterogeneous echotexture. In contrast, when we compare tumors with similar size, no differences are found; thus, the difference between groups may be due to the size of the tumors because the SG group is composed of smaller tumors, which have a higher prevalence of hypoechoic echogenicity and homogeneous echotexture.

Our results revealed a relationship between the definition of tumor margins in the initial stages of tumor development and growth rate. The SG tumors presented circumscribed margins in all examinations until day 12 after induction, while the FG tumors presented indistinct margins on the same day in 45.8% of examinations, but there was no difference when we compared ISV. Therefore, the difference between days until day 12 may be caused by the difference in tumor volume between groups or because the number of tumors is insufficient to obtain statistically significant difference.

Previous studies did not report differences in the definition of tumor contours between benign and malignant tumors (19, 20, 31, 34), but a study showed differences between high- and low-grade tumors in human invasive ductal carcinoma where the group of neoplasms with higher grade had a higher proportion of undefined margins (32). In contrast, these differences were not found in canine mammary carcinomas (33)

In this study, ESC was identified by Doppler ultrasonography like moderately vascularized tumors that presented diffuse blood flow distribution in the initial stages of growth and peripheral blood flow distribution in advanced stages. This change in the pattern of flow distribution is more evident in FG tumors.

We observed that the percentage of images with vascular flow and VI were higher in the FG group, especially in the early stages of growth, even when we compared similar volumes. There is no unanimous conclusion about the relationship between the quantity of vascular flow observed by ultrasonography and malignancy; some authors did not find statistically significant differences (19, 31), while others reported greater vascularization in benign (20) or malignant neoplasia (35, 36). This difference in results is because of the wide variety of breast tumors that may have different vascularization patterns. For example, a study showed that carcinomas have greater vascularization than sarcomas (11). In our study, the comparison was between the same type of malignant tumor, and significant differences were observed between the groups with higher and lower growth. This difference could be explained by considering the biology of ESC.

ESC has an angiogenesis-dependent growth (37). Initially, solid growth is dependent on passive diffusion of oxygen from the surrounding stroma; however, as the tumor lesion grows to 1–2 mm, core cells start to accumulate hypoxia-inducible factors, which trigger the angiogenic switch (38). Vascular flow is important because the volume of the tumor is small, and it is essential for rapid growth; therefore, tumors that achieve more angiogenesis may grow faster (37). In a study quantifying tumor perfusion by acoustic angiography, was observed that an animal of the untreated control group had small tumor growth and exhibited poor perfusion within the tumor compared to the other animals of the same group (39). In contrast, when we analyze the evolution of VI over time, we observe that the FG group has a higher initial VI but tends to decrease over time. This is because those tumors initially achieve greater vascularization and grow faster, but they grow rapidly and cannot maintain adequate angiogenic index to vascularize the entire tumor volume with mature and flowing vessels (40); thus, the number of vessels observed by ultrasound per area is smaller. In addition, over time, there is a tendency toward mixed echogenicity that may reflect the appearance of necrotic areas, among other things, which also reduces vascularized areas. In slow growing tumors, the VI remains stable because it has more time to achieve mature vascularization.

Previous studies comparing vascular distribution with malignancy did not find unanimous conclusions. Some authors did not report differences between vascular flow pattern and tumor type group (20, 31), while others reported differences in which benign tumors tend to show peripheral patterns, while malignant tumors show mixed patterns (19). This study was conducted with the same type of malignant tumor; in the FG group, peripheral pattern was the most frequently observed, while in the SG group, the most frequent pattern was diffuse, but no statistically significant differences were observed. Considering the histology and biology of ESC, tumors with greater volume tend to have a heterogeneous structure, with necrotic internal parts, and have immature vessels. For this reason, the main vascularization is in the peripheral regions.

In the present study, higher SV was observed in the FG group; in mammary neoplasms in human and animals, this has been described as indicator of malignancy (20, 41). This may be related to the presence of tortuous vascular networks in malignant tumors, especially in tumors that grew rapidly. The ultrasonographic differences between the FG and SG groups are related to vascularization; therefore, it shows the influence of neoplastic vascularization in neoplastic nutrition and growth index.

Non-invasive image techniques for quantitative tissue characterization and assessing microvasculature are being increasing used in experimental oncology. Quantitative ultrasound parameters demonstrated sensitivity to microstructural changes in tissue, offering the possibility of estimating the cell death rate and assess response to treatments (42). Recently, DCE-US display a good performance assessing microvascular dispersion and perfusion, showing that it could be an important tool to cancer diagnosis (9, 10). We have perspectives to apply this technique to ESC in future studies.

## Data Availability Statement

The raw data supporting the conclusions of this article will be made available by the authors, without undue reservation.

## Ethics Statement

The animal study was reviewed and approved by Comissão de Ética no Uso de Animais (CEUA)/Universidade Federal de Goiás.

## Author Contributions 

CC designed, executed experimental work, performed ultrasound exams and data analyses and wrote the manuscript. MM contributed to data analyses, discussion, review the final manuscript and followed publication process. NB and AB designed experimental work and project co-director. ES provided the tumor models and contributed to discussion. All authors contributed to the article and approved the submitted version.

## Conflict of Interest

The authors declare that the research was conducted in the absence of any commercial or financial relationships that could be construed as a potential conflict of interest.

## Abbreviations

DT, Doubling Time; DCE-US, Dynamic Contrast-Enhanced Ultrasound; ESC, Ehrlich Solid Carcinoma; FG, Fast Growth Group; ISV, Initial Similar Volumes; LSV, Largest Similar Volumes; PI, Pulsatility Index; SG, Slow Growth Group; SGR, Specific Growth Rate; SV, Peak Systolic Velocity; VI, Vascularity Index.
